# The postoperative cognitive dysfunction induced by central inflammation with possible involvement of the gut-brain axis

**DOI:** 10.1016/j.clinsp.2022.100104

**Published:** 2022-09-19

**Authors:** Chuantao Lin, Jing Wang, Yuping Wang, Chanjuan Chen, Xiang Gao

**Affiliations:** Department of Anesthesiology, Fujian Maternity and Child Health Hospital; College of Clinical Medicine for Obstetrics & Gynecology and Pediatrics, Fujian Medical University, Fujian, China

**Keywords:** Postoperative cognitive dysfunction, Tibial fracture, CCL11, Gut-brain axis, POCD, Postoperative cognitive dysfunction, CCL11, Eotaxin contains eotaxin-1, NOX1, nicotinamide adenine dinucleotide phosphate-oxidase 1, ROS, reactive oxygen species, GBA, gut-brain axis

## Abstract

**Background:**

Postoperative cognitive dysfunction is widely recognized as severe postoperative central nervous dysfunction and has a significant impact on the ’patient's physical and mental health.

**Methods:**

Postoperative models of tibial fracture in aged rats were established, including the control group, model group, CCL11 protein injection group, and saline injection group. Morris water maze test was used to detect the behavioral characteristics of rats. Enzyme-Linked Immunosorbent Assay was used or determine the content of CCL11 and CXCL10. Immunofluorescence staining was used to detect the distribution of CD14+CD163+macrophages in colon tissues and CD11b+CCR3+microglia cells in hippocampal tissues. Western blot analyzed NOX1 and STAT3 expression in hippocampus tissues.

**Results:**

Water maze test results confirmed severe cognitive impairment in CCL11 rats. The content of CCL11 and CXCL10 in the CCL11 group was much higher than that of the model group. The distribution of macrophage and microglia cells in the CCL11 model group was greater than that in the model group and the saline group. The expression of NOX1 and STAT3 in the CCL11 group was higher compared with the model group.

**Conclusion:**

Abnormal macrophage function and excessive CCL11 secretion were observed in the rats with lower limb fractures after surgery. Postoperative central inflammation in rats with lower limb fracture induced postoperative cognitive dysfunction through the gut-brain axis molecular mechanism.

## Highlights

• POCD has a significant impact on patients' physical and mental health.
• Postoperative models of tibial fracture in aged rats were established.
• Postoperative central inflammation in rats with lower limb fracture induced POCD through the GBA molecular mechanism.

## Introduction

With the improvement of medical level, more and more diseases will be treated by surgery, while cognitive dysfunction after surgery has a significant impact on a patient's physical and mental health, quality of life, and social regression. It has been reported that 50% of all the elderly individuals in the world are estimated to perform at least one surgical procedure. Studies indicate that approximately a quarter of the elderly with major surgery will have an identifiable fall in cognition, and 50% of these patients will suffer a permanent dysfunction.^1^ Postoperative Cognitive Dysfunction (POCD) is recognized as acute/persistent postoperative central nervous dysfunction, and affects an orientation, attention, perception, consciousness, and judgment that develop after surgery.^2^ According to a recent study, about 36.6%‒41.4% of patients have POCD symptoms at the time of discharge.^3^ POCD symptoms occur in all ages and can continue from days to years. POCD is characterized by acute or persistent declines in attention, concentration, learning, and memory, or other impairment of brain function.^4^

In recent years, neuroinflammation induced by surgical factors is considered to be an essential cause of POCD.^5^ Related studies have shown that chemokines (eotaxin) may induce cognitive dysfunction. Eotaxin contains eotaxin-1 (CCL11), eotaxin-2 (CCL24) and eotaxin-2 (CCL26). Among these, CCL11 can bind to CCR3 receptors on the macrophage in the central nervous system to exert the release of reactive oxygen species, which mediates the cytotoxic effect in the brain neurons and results in memory loss in mice.^6^ Therefore, in-depth research of CCL11 is conducted to further understand the pathogenesis of POCD, thus providing a potential direction for the effective treatment of POCD.

CCL11 is a CC chemokine and an eosinophilic chemokine that regulates tissue eosinophilia in the context of inflammation, asthma, dermatitis, and parasitic infections. CCL11 can significantly promote reactive oxygen species release to damage the neurons in adults, leading to cognitive ability decline.^7^ CCL11 is the main effector molecule of microglial cells in the central nervous system. CCL11 promotes microglial cell migration and the production of reactive oxygen species. CCL11 significantly promoted the migration of microglia and induced microglial production of reactive oxygen species by upregulating nicotinamide adenine dinucleotide phosphate-oxidase 1 (NOX1). In microglia, ROS are mainly produced by NOX1.^8^ In addition, CCL11-mediated microglial cell produces Reactive Oxygen Species (ROS) to enhance excitotoxic neuronal death, which is believed to be the pathogenic mechanism of neurodegenerative diseases in various nervous system diseases.^8^

Studies have confirmed that alteration of intestinal flora will cause intestinal immune disorders, inflammatory factor release, and abnormal macrophage function. Activation of intestinal macrophages has a close relationship with disease severity.^9^ A number of evidence from animals and humans reveal a complex and persistent interaction of the intestine and brain, which not only establishes the regulatory dynamic balance of the gastrointestinal tract, but can also have multiple effects on emotion, activity, and cognitive function, known as the Gut-Brain Axis (GBA). The effect of GBA is to regulate and integrate the functions of the gastrointestinal system, such as intestinal reflex, intestinal permeability, immune activation, intestinal endocrine signal, and the influence on the emotional and cognitive abilities of the brain.^10^

It is well known that gastrointestinal dysfunction is common in old patients with lower limb fractures after surgery, mainly due to the following reasons: 1) Post-traumatic stress response; 2) Degenerative changes of gastrointestinal function in the elderly; 3) Anesthetic drugs. This study intends to observe the changes in cognitive function in the aged rat model of tibial fracture by behavioral methods and immunohistochemical methods. Meanwhile, the hippocampal activation of NOX1 was detected, revealing the theory that CCLL11 acts on the nervous system through the gut-brain axis, and providing an experimental basis for the targeted regulation of CCL11 to improve the cognitive level of POCD patients.

## Materials and methods

Aged SD rats were purchased from Junke Animal Technology Company (Nanchang, China). The rats were 40 weeks of age and were adaptively fed for 2 weeks under an SPF level environment, 25°±2°C, with humidity of 50%. All the procedures for rats were agreed upon with the Ethics Committee of Fujian Maternity and Child Health Hospital, Affiliated Hospital of Fujian Medical University on August 3, 2018. Institutional Animal Care and Use Committee (IACUC) approval number 180086. CCL11 protein, Enzyme-Linked Immunosorbent Assay (ELISA) kit were purchased from sigma (USA); 3% isoflurane and PBS were purchased from Gibco (Shanghai, China); SDS-PAGE electrophoresis was purchased from Thermo Fisher (USA); Rabbit anti-rat primary antibody NADPH Oxidase 1 (NOX1) and Signal Transducer and Activator of Transcription 3 (STAT3) were purchased from innochem; Morris water maze was purchased from Nolds Ethovisin 3.0 (Netherlands). Human CCL11 protein (10474-H07E-50, 50 μg) was purchased from Innochem. CXCL10 (D5L5L) Rabbit (14969S, 100 μL, CST) was purchased from Innochem.

### Methods

#### Establishment of a postoperative model of tibial fracture in aged rats

48 aged SD rats were fed the standard granulated feed and free water, then randomly divided into 4 groups. There were 12 rats in every group: A-normal group (healthy rat); B-model group; C-CCL11 protein injection group of model rats (CCL11 group); D-saline injection group of model rats (saline group). The method of the postoperative model of tibial fracture in aged rats was improved according to the previous method.^11^^,^^12^ The rats were anesthetized by using 3% isoflurane and the right lower limb of the rat was disinfected after the skin was peeled off. A longitudinal incision of about 0.5 cm was made under the right knee joint of the rats. A 22G/041 mm intramedullary fixation needle was inserted from the kneecap tendon, and the muscle and fascia were separated at the middle and upper 1/3 of the tibia. After the tibial diaphysis was sawed with a saw blade, the tibial surface was irrigated with 0.9% normal saline immediately. Then the intramedullary fixation needle was completely inserted and aligned. A rat model of right tibial shaft fracture was established by sutured incisions layer by layer. After the operation, the rats were raised in cages and given regular water and a diet in a clean environment, so they could move freely. The rats in the C group were intraperitoneally injected with CCL11 protein (100 ng/mL, 1 mL), once a day. While the rats in the D group were intraperitoneally injected with saline (0.9%, 1 mL). All rats were treated for one week.

#### Morris water maze test

The morris water maze test uses a 160 cm diameter pool with a height of 50 cm. The water depth is 31 cm and the temperature keeps at 23°C.^13^ After CCL11 treatment for 5 days, the rats were put into the water, then their heads faced the wall. All rats were randomly selected for one position for starting the research. Recorded the time that the rats took to find the underwater platform (s). In previous training, if the time exceeded the 60s, the rats were guided to the platform. Let the rats stay on the platform for 10s. Every rat was trained 4 times a day, with an interval of 15 to 20 min between the two training sessions, and the training lasted for 5 days.

#### Detection of the CCL11 and CXCL10 content

The rats were sacrificed and fixed on the operating table supine to make the abdominal aorta fully exposed. Blood was collected from the abdominal aorta by vacuum vascular collection. Enzyme-Linked Immunosorbent Assay (ELISA) kit was operated according to the instructions, and the results were read on the microplate reader to analyze the content of inflammation-related factors CCL11 and CXCL10.

#### Distribution of CD14+CD163+ macrophages in colon tissues

The rats were sacrificed to take the colon tissues from the anal doorway 5 cm away, then the tissues were fixed with a 10% neutral formaldehyde solution. The distribution of CD14+CD163+macrophages in colon tissues was detected by immunofluorescence staining. Immunofluorescent staining: the tissue was placed at 65°C for 30 min, and then soaked in xylene for 15 min. The tissues were added to the primary antibody and incubated at 4°C overnight after being washed for 10 min. Fluorescent secondary antibody continuously incubated at 25°C for 1h when they were washed with 0.02M PBS 3 times. The tissues were sealed by anti-quench sealant DAPI (1:500) and photoed by fluorescence microscopy.

#### Distribution of CD11b+CCR3+microglia cells

The rats were anesthetized by using 3% isoflurane. The blood was irrigated with 200 mL saline by left ventricular perfusion and then perfused with 4% paraformaldehyde for 5‒10 min. The brain was immediately taken after perfusion, and the hippocampus was separated to place in 4% paraformaldehyde to fix for 4h, then the brain was transferred to 30% sucrose solution until the bottom sank. Coronal frozen sections with a thickness of 50 μm were prepared and collected in 0.1 moL/L phosphate buffer. Immunofluorescence staining was used to analyze CD11b+CCR3+ microglia cells in hippocampus tissue.

### Analysis of NOX1 and STAT3 expression

Total proteins of hippocampus tissue were extracted, After SDS-PAGE electrophoresis, membrane transfer, and antibody incubation, the protein expression of NOX1 and STAT3 was determined by western blot. Rabbit anti-rat primary antibodies NOX1 and STAT3 (1:500) were added and incubated at 4°C overnight. TBST was washed 4 times, then HRP labeled sheep anti-rabbit secondary antibody (1:5, 000) was added and incubated at 37°C for 1h. TBST was washed 4 times. Bio-rad electrophoretic gel imaging analyzer was used for collection and gray value measurement.

### Statistical analysis

After data collection and summary, statistical professionals of the Mathematical Statistics Teaching and Research Office of the University were entrusted to be responsible for data analysis and processing, and SPSS20.0 statistical software package was adopted for data statistical analysis. Results were expressed as mean±SEM. Multigroup comparisons of the means were carried out by one-way analysis of variance (ANOVA) test with post hoc contrasts by Student-Newman-Keuls test. The statistical significance for all tests was set at p < 0.05.

## Results

### Morris water maze test

After modeling, the behaviors of rats changed and the activities of rats decreased, but the amount of food and water was basically normal. The total swimming distance in the model group, CCL11 group, and saline group was significantly shorter than that of the normal group, especially the CCL11 group. There was a significant difference, *^⁎⁎^*p<0.01, as shown in Table 1. Average swimming speed in CCL11 group was lower compared with the model group, and there was an obviously significant difference, *^#^*p<0.05. Times of platform passes in the CCL11 group were lower compared with the model group, and there was an obviously significant difference, *^#^*p<0.05. There was no significant difference between the model group and the saline group in total swimming distance, average swimming speed, and times of platform passes.

**Table 1:** Rats Morris water maze test in different groups (X ± SD, n = 48).

Group	Total swimming distance (m)	Average swimming speed (mm/s)	Times of platform passes
Normal group	7.73 ± 0.23	86.59 ± 8.33	6.92 ± 0.77
Model group	5.21 ± 0.66*	67.59 ± 8.14*	3.65 ± 0.68^a^
CCL11	3.51 ± 0.87^a^^,^^b^	54.53 ± 6.34^a^^,^^b^	2.53 ± 1.21^a^^,^^b^
Saline	5.22 ± 0.91^*,^^b^	72.66 ± 7.62	3.99 ± 0.74^a^

Note:

a p < 0.01, compared with NC group.

b p < 0.05, compared with model group.

The Morris water maze test is a spatial learning-memory model designed by British psychologists in the 1970s and applied to the study of brain mechanisms. This kind of spatial recognition learning relies on the complex cognitive processes of information acquisition, recall, evaluation, comparison, and reasoning. The results of water maze test confirmed that the rats in the CCL11 group had severe cognitive impairment compared with the normal group and the model group.

### Content of inflammatory factors CCL11 and CXCL10

**Fig. 1:**
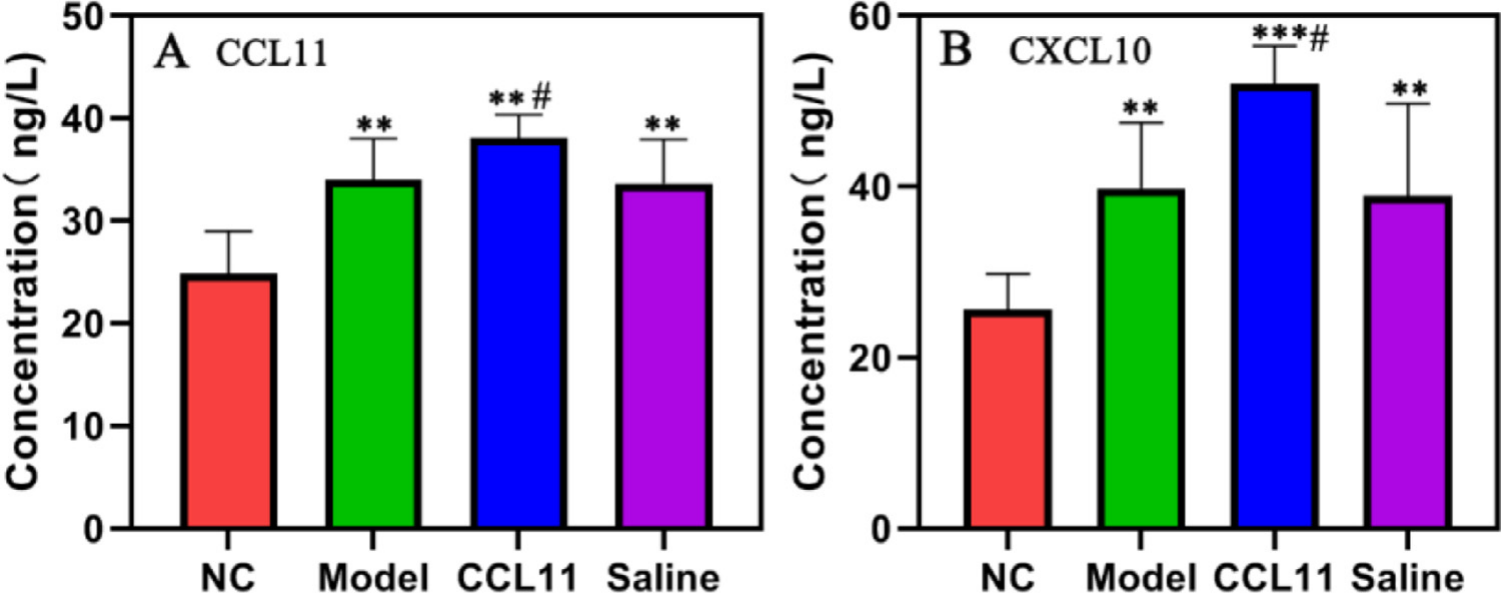
The content of inflammatory factors CCL11 and CXCL10. (A) Concentration of CCL11 (ng/L); (B) Concentration of CXCL10 (ng/L) in four groups. *^⁎⁎⁎^*p < 0.001, *^⁎⁎^*p < 0.01, compared with NC group; *^#^*p < 0.05, compared with model group.

The content of inflammatory factors CCL11 and CXCL10 in the model group was shown in Fig. 1. The model group, CCL11 group and saline group showed much higher CCL11 and CXCL10 concentrations than that of the normal group, ^⁎⁎^p < 0.01. Content of CCL11 and CXCL10 in the CCL11 group was higher compared with the model group, ^#^p < 0.05. There was no significant difference between the model group and the saline group.

### Distribution of CD14+CD163+ macrophages in intestinal tissue

**Fig. 2:**
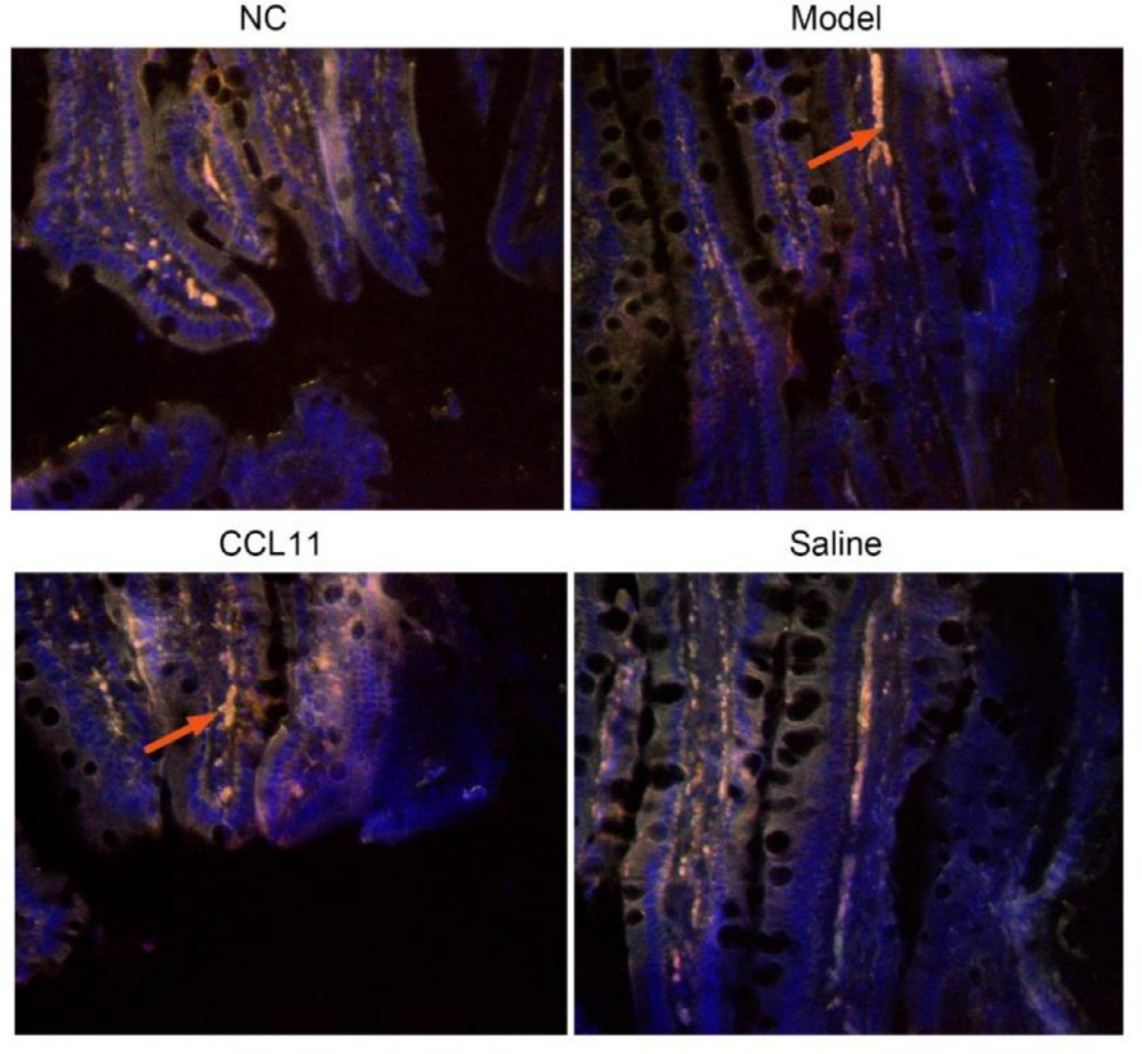
Immunofluorescence distribution of macrophages in intestinal tissue. CD163 is a glycoprotein expressed on the membrane of macrophages, which was labeled in red arrow. *p < 0.05 compared with model group.

Soluble leukocyte differentiation antigen 14 (CD14) is the anchor protein of cell membrane and the main receptor of endotoxin. It activates macrophages to release inflammatory factors and triggers a cascade reaction, which is an important indicator of inflammation evaluation.^14^ CD163 is a glycoprotein expressed on the membrane of monocytes and macrophages and is an important serum marker for early infection and inflammation.^15^ CD163 is a transmembrane protein expressed and activated by monocytes and macrophages, which is significantly increased when the body is infected and macrophages increase after inflammation.^16^ As shown in the arrow, the distribution of macrophages in the CCL11 group was larger than that in the model group and the saline group. Both the model group and the saline group were larger than that in the normal group, as shown in Fig. 2.

### CD11b+CCR3+microglia cells distribution in hippocampal tissues

**Fig. 3:**
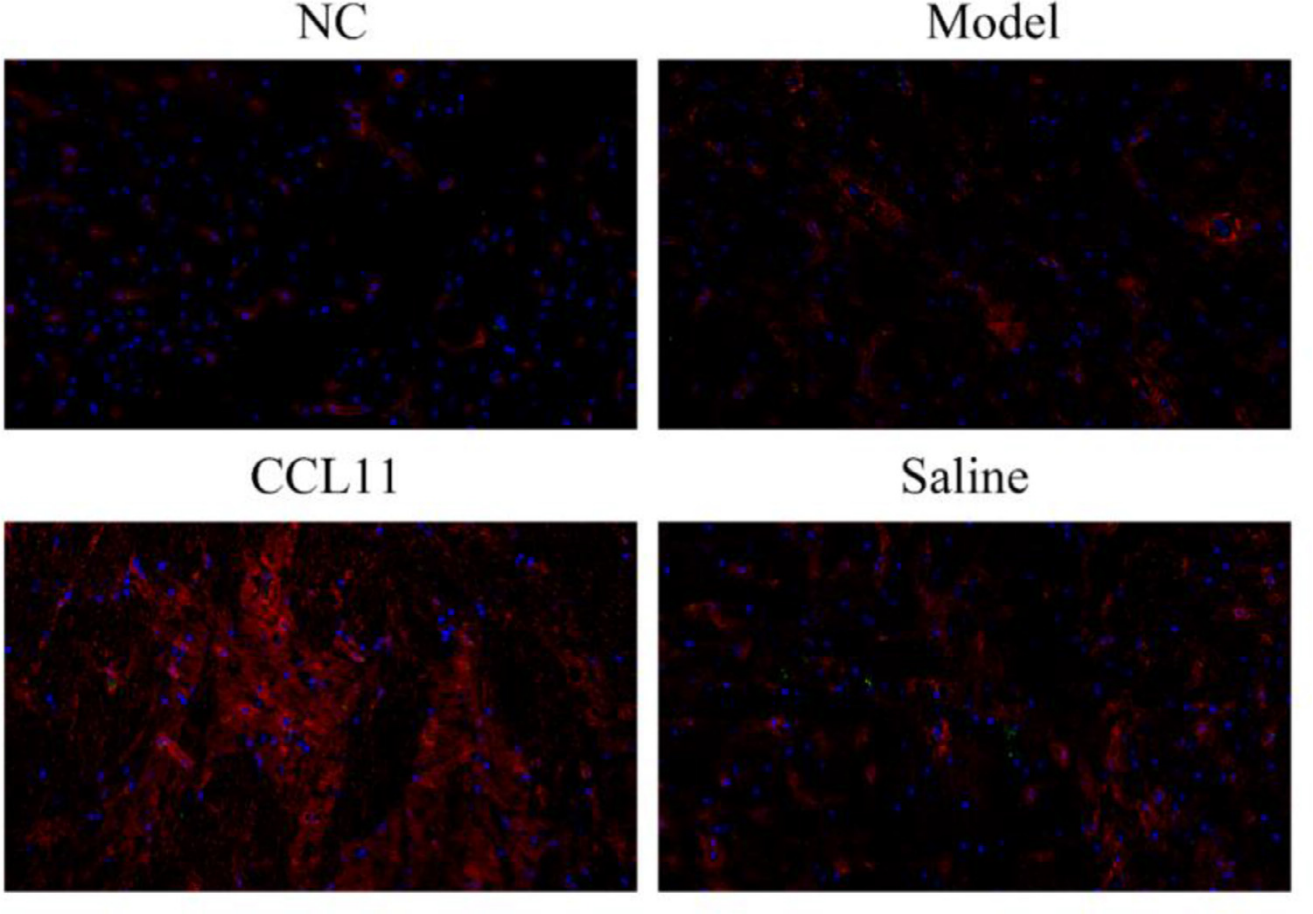
Immunofluorescence staining analysis of CD11b+CCR3+microglia cells distribution in hippocampal tissues. The microglia cells in the hippocampus were labeled fluorescent red. *p < 0.05 compared with model group.

Microglial cells, as an important defense barrier of the central nervous system and an important component of the innate immune system, play an vital role in pathogen elimination and mediating inflammatory factor release. Dysfunction of the microglial cell is involved in the development of cognitive impairment. The distribution of microglia cells in the hippocampus was analyzed by immunofluorescence staining, as shown in Fig. 3. Microglia cells in the hippocampus were labeled blue with hochest, and the distribution of microglia cells in the CCL11 group was larger than that of the model group and the saline group.

### Western blot analysis of NOX1 and STAT3 expression

**Fig. 4:**
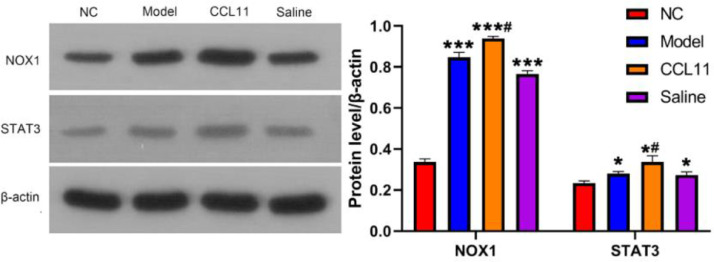
Protein levels of NOX1 and STAT3 in different groups determined by western blot (Mean ± SD, n = 3). *^###^*p < 0.001, *^#^*p < 0.05 compared with normal group; *p < 0.05 compared with model group.

The expressions of NOX1 and STAT3 proteins in the hippocampus of rats were shown in Fig. 4. The expression of NOX1 and STAT3 proteins in the model group, CCL11 group, and saline group were much higher than that of the normal group, ^⁎⁎⁎^p < 0.001. The expression of NOX1 and STAT3 in the CCL11 group was higher compared with the model group, and there was a significant difference, ^#^p < 0.05. However, there was no significant difference between the model group and the saline group, p > 0.05.

## Discussion

The highly selective Chemotaxis of Eotaxin (CCL11) of eosinophils is the most important material. CCL11 is also a strong agonist for eosinophilic respiratory bursts. Macrophages and eosinophils are the main sources of CCL11, which increase the production of CCL11. CCL11 can initiate eosinophils, lymphocytes, and Th2 subsets, promote the release of histamine by basophils, induce the growth of mast cells, and aggravate inflammation.^17^ The rats in the CCL11 model group appeared in inflammatory reactions, which indicated that CCL11 mediated the production of ROS in microglia cells and enhanced the excitotoxic neuronal death to induce cognitive dysfunction.

In human and mouse studies, many studies have identified macrophages is the primary source of CCL11. CD14 is an important mediator that regulates the effect of lipopolysaccharide and non-medullary cells, has an essential role in regulating the activity of lipopolysaccharide, and sensitizing lipopolysaccharide cell effect in vivo, and CD14 has been found to be positively correlated with the severity of disease infection.^18^ Lipopolysaccharides can up-regulate the expression of CD14, increase the shedding of CD14 on the surface of mononuclear macrophages, and produce an inflammatory cascade that leads to increased inflammation in the body.^19^ CD163 is a novel anti-inflammatory mediator that activates the secretion of mononuclear macrophages. When the body is infected, membranous CD163 is shed into the serum through the matrix metalloprotein pathway, and the level of CD163 in the serum is significantly increased.^20^ This study found that the level of CD163 in the model group, CCL11 group and normal saline group was obviously higher than that in the normal group. This was mainly due to the lower extremity fracture of the elderly rats, which resulted in the imbalance of intestinal flora and appeared to abnormal function of intestinal macrophages. Intestinal flora imbalance will cause intestinal immune disorders, inflammatory cytokine release, and macrophage dysfunction. The activation of intestinal macrophages was positively correlated with disease severity.

Microglia cells as an important defense barrier of the central nervous system, account for about 5%‒10% of the total number of brain cells and play an important role in maintaining homeostasis in the brain.^21^^,^^22^ Microglia cells are also an important component in the system of innate immunity, which plays an important role in pathogen elimination and mediating inflammatory factor release. They are also considered indispensable cells in cognitive processes such as learning and memory. Microglia-mediated neuroimmune regulatory dysfunction plays an important role in cognitive impairment and other central neurodegenerative diseases. Microglia cell is a kind of highly malleable cell that differentiates into two activated phenotypes based on specific microenvironmental signals in the brain:^23^ Classical activation (M1) and alternative activation (M2). M1 mainly produces a lot of inflammatory factors, causes inflammatory reactions and the degeneration and necrosis of dopaminergic neurons, and promotes POCD.^24^ In related studies of cognitive function, overactivation of microglia leads to the production of inflammatory cytokines, and persistent and chronic neuroinflammation of these factors may result in nerve cell dysfunction and death.

**Fig. 5:**
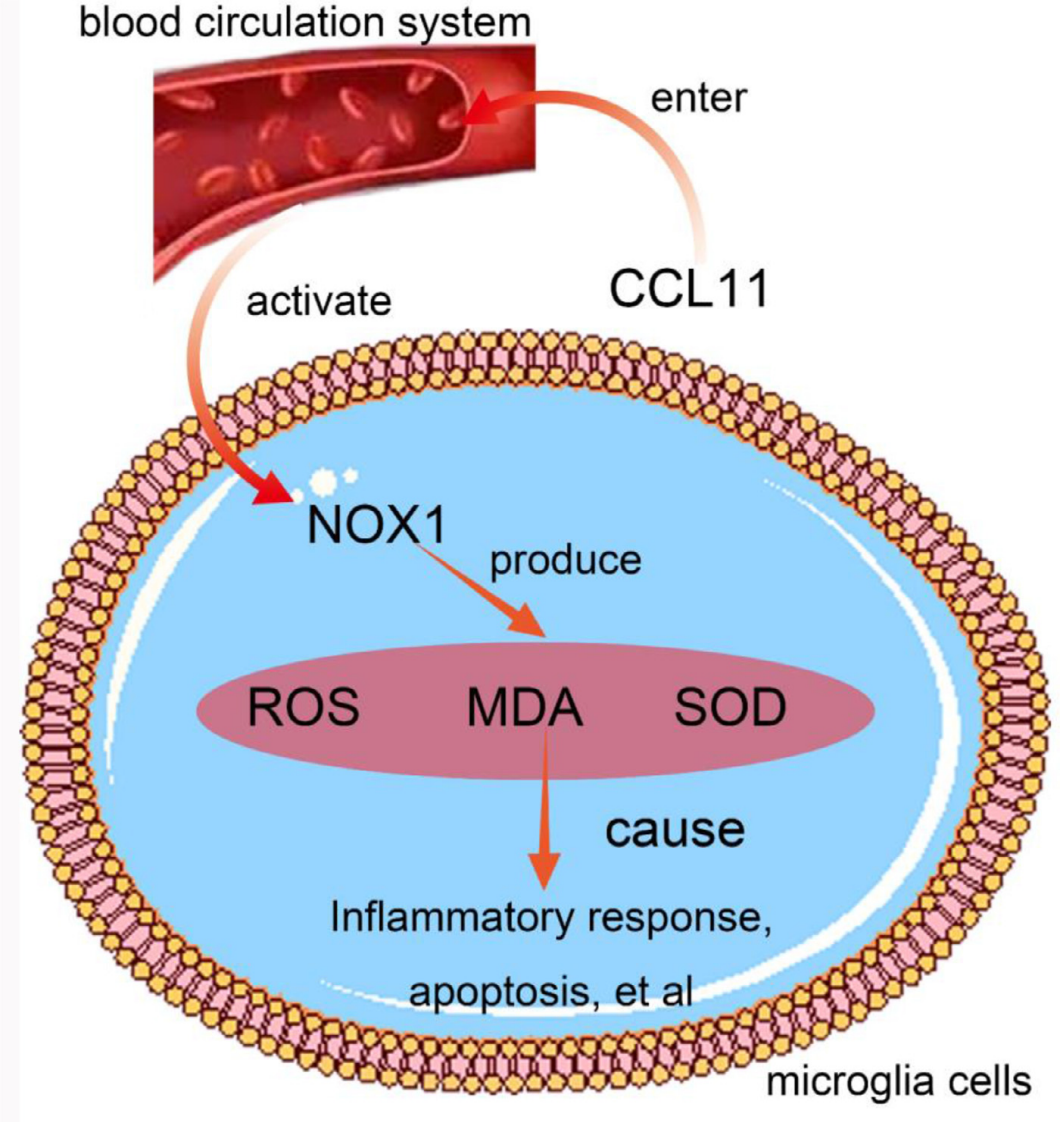
Schematic diagram of CCL11 promoted the migration of microglia and induced microglial production of reactive oxygen species by upregulating NOX1.

In microglia cells, ROS is mainly produced by NOX1 and NOX2. CCL11 results in significant upregulation of NOX1, but not of NOX2. Further studies have shown that CCL11-mediated neurotoxicity of microglia depends on NOX1 activation. In fact, upregulation of microglia NOX1 has been observed in a variety of neurological diseases, such as multiple sclerosis and Parkinson's disease. Nicotinamide adenine dinucleotide phosphate oxidases (NOX1) are the main source of ROS.^25^ Under normal conditions, the levels of H_2_O_2_ and superoxide anion produced by NOX1 are relatively low, but NOX1 acutely activates under various pathological conditions such as ischemia, hypoxia, hyperglycemia, and hypertension.^26^ In the rat model with lower limb fracture after surgery, CCL11 enters the blood circulation system and activates reactive oxygen species produced by NOX1 in microglia cells of the hippocampus. NOX1 causes an increase in reactive oxygen species, Malondialdehyde (MDA) content, and reduces the activity of Superoxide Dismutase (SOD). All of this causes an inflammatory response, hypertrophy, proliferation, apoptosis, migration and dysfunction of cells,^27^^,^^28^ resulting in neuronal dysfunction and POCD,^29^ as shown in Fig. 5.

## Conclusion

Abnormal macrophage function and excessive CCL11 secretion were observed in the rats with lower limb fractures after surgery. Postoperative central inflammation in rats with lower limb fracture induced postoperative cognitive dysfunction through the gut-brain axis molecular mechanism.

## Authors' contributions

Chuantao Lin, Jing Wang, Yuping Wang, Chanjuan Chen, Xiang Gao wrote the manuscript and conducted most of the experiments, Chuantao Lin, Jing Wang, Yuping Wang, Chanjuan Chen conducted the experiment, collected, and analyzed the data, Xiang Gao designed the study, and all authors approved the submission.

## Funding

2019 Fujian Provincial Maternity and Child Health Hospital Science and Technology Innovation Startup Fund (YCXM19-19).

## Conflicts of interest

The authors declare no conflicts of interest.
